# Supplementary material for: Identification of leaf rust resistance loci in hard winter wheat using genome‐wide association mapping
Source: Plant Genome. 2025 Jan 5;18(1):e20546. doi: 10.1002/tpg2.20546 (PMC11700927; doi:10.1002/tpg2.20546)
Supplement: Supplementary file 2 — Supplemental Figure S1. Pearson correlations between reactions of 459 hard winter wheat genotypes to five Puccinia triticina isolates tested at the seedling stage. Supplemental Figure S2. Pearson correlations between leaf rust reactions of hard winter wheat genotypes in field environments. Supplemental Figure S3. Chromosome‐wise distribution of 9,858 SNP markers in 459 hard winter wheat genotypes selected from the 2021 and 2022 NRPN, SRPN, and RGON. Supplemental Figure S4. Density of SNP markers (n = 9858 SNPs) per 1Mb window on wheat chromosomes in a set of 459 hard winter wheat genotypes selected from the 2021 and 2022 NRPN, SRPN, and RGON. Supplemental Figure S5. Scatter plot showing linkage disequilibrium (LD) decay across the genome. Supplemental Figure S6. Scatter plot showing linkage disequilibrium (LD) decay in sub genomes A, B, and D. Supplemental Figure S7. Percentages of 732 wheat genotypes originated from the 2021 and 2022 NRPN, SRPN, and RGON carrying different number of resistant alleles of the 59 significant SNPs associated with leaf rust response. Supplemental Figure S8. Violin plots show leaf rust response distributions of genotypes carrying resistant and susceptible alleles of nine SNPs that can be useful for marker‐assisted selection. [file TPG2-18-e20546-s002.docx]

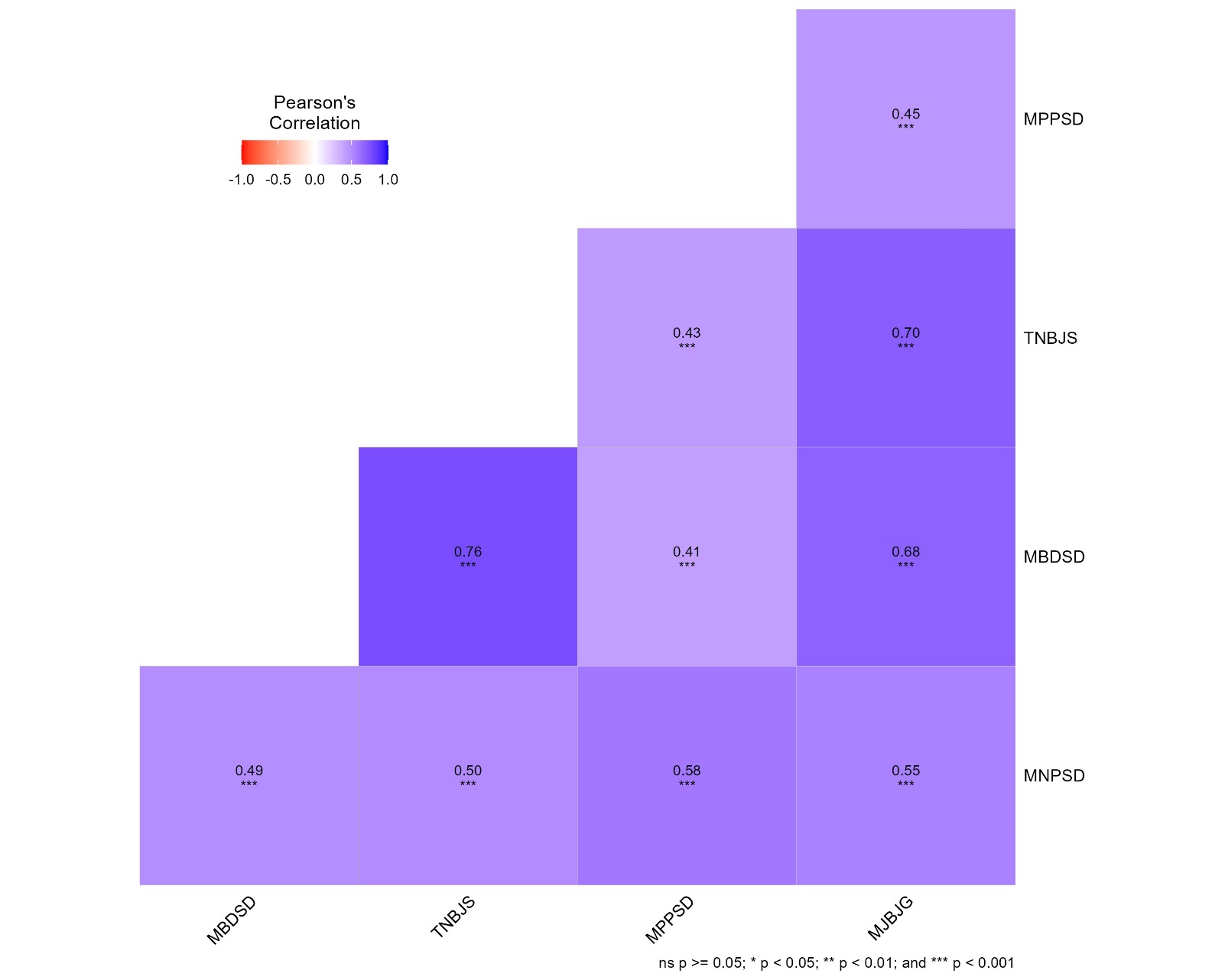


**Supplemental Figure S1**. Pearson correlations between reactions of 459 hard winter wheat genotypes to five *Puccinia triticina* isolates tested at the seedling stage.


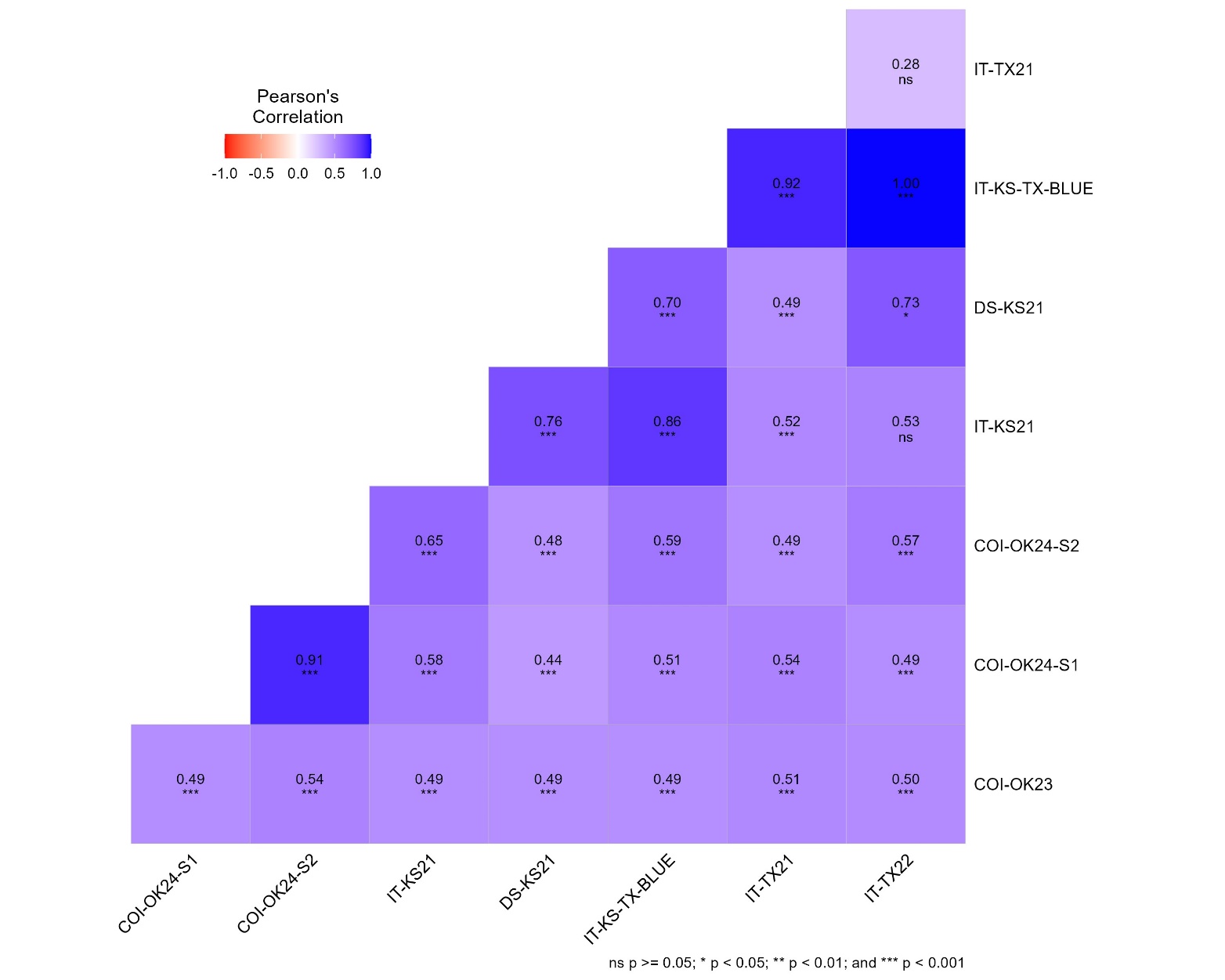


**Supplemental Figure S2**. Pearson correlations between leaf rust reactions of hard winter wheat genotypes in field environments. COI-OK23: coefficient of infection of 2021 and 2022 NRPN, SRPN and RGON genotypes (n = 459) tested at the adult plant stage in Oklahoma in 2023; COI-OK24-S1 & COI-OK24-S2: first and second scoring of coefficient of infection of 2021 and 2022 NRPN, SRPN, and RGON genotypes (n = 459) tested at the adult plant stage in Oklahoma in 2024, respectively; IT-TX21: ITs of 2021 RGON genotypes (n = 300) tested at the adult plant stage in Texas in 2021; IT-KS21: ITs of 2021 NRPN, SRPN, and RGON genotypes (n = 377) tested at the adult plant stage in Kansas in 2021; DS-KS21: Disease severity of 2021 NRPN, SRPN, and RGON genotypes (n = 377) tested at the adult plant stage in Kansas in 2021; IT-KS-TX-BLUE: best linear estimates for ITs of 681 genotypes (IT-KS-TX-BLUE) across three field environments IT-TX21, IT-TX22, and IT-KS21.


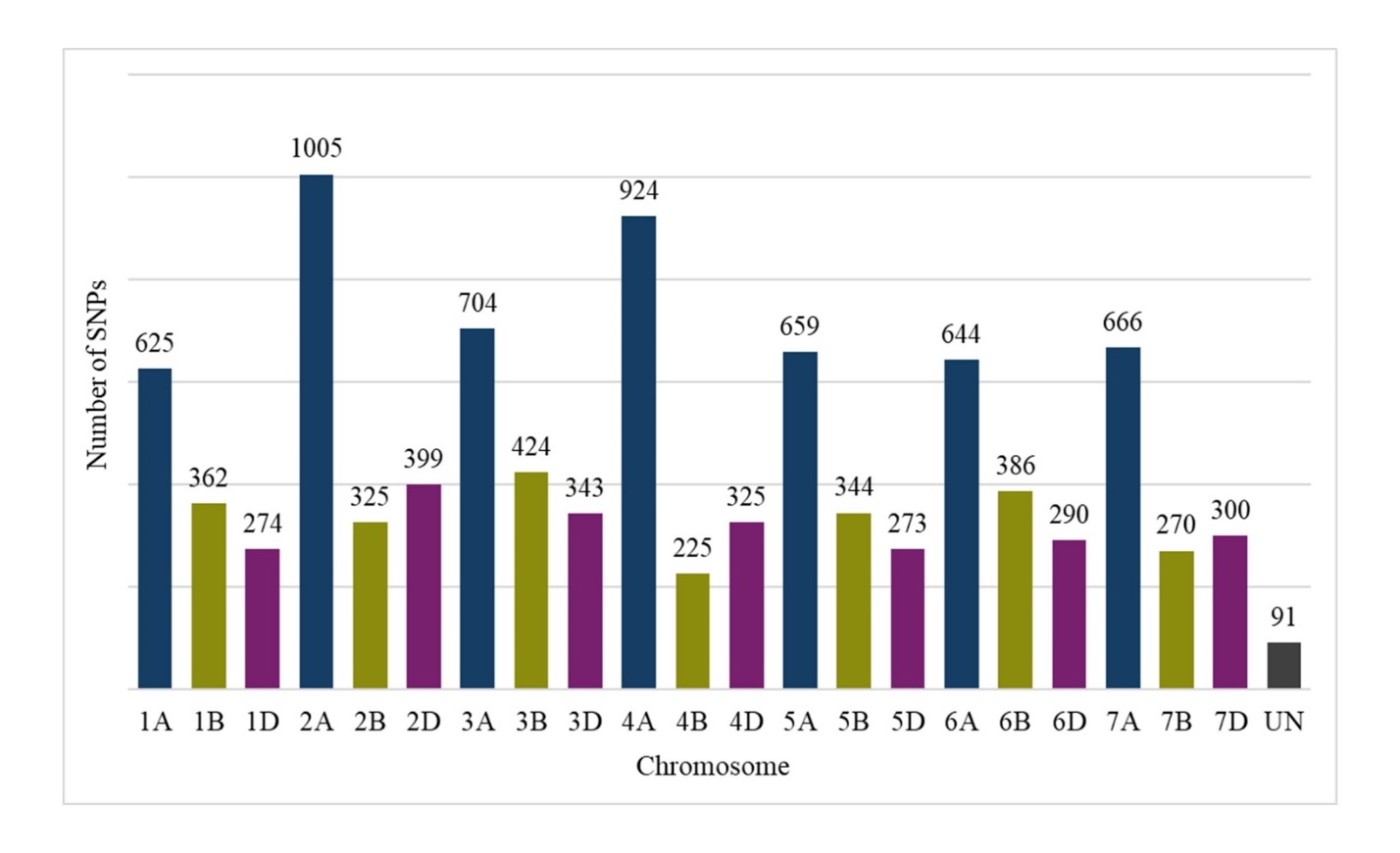


**Supplemental Figure S3.** Chromosome-wise distribution of 9,858 SNP markers in 459 hard winter wheat genotypes selected from the 2021 and 2022 NRPN, SRPN, and RGON.


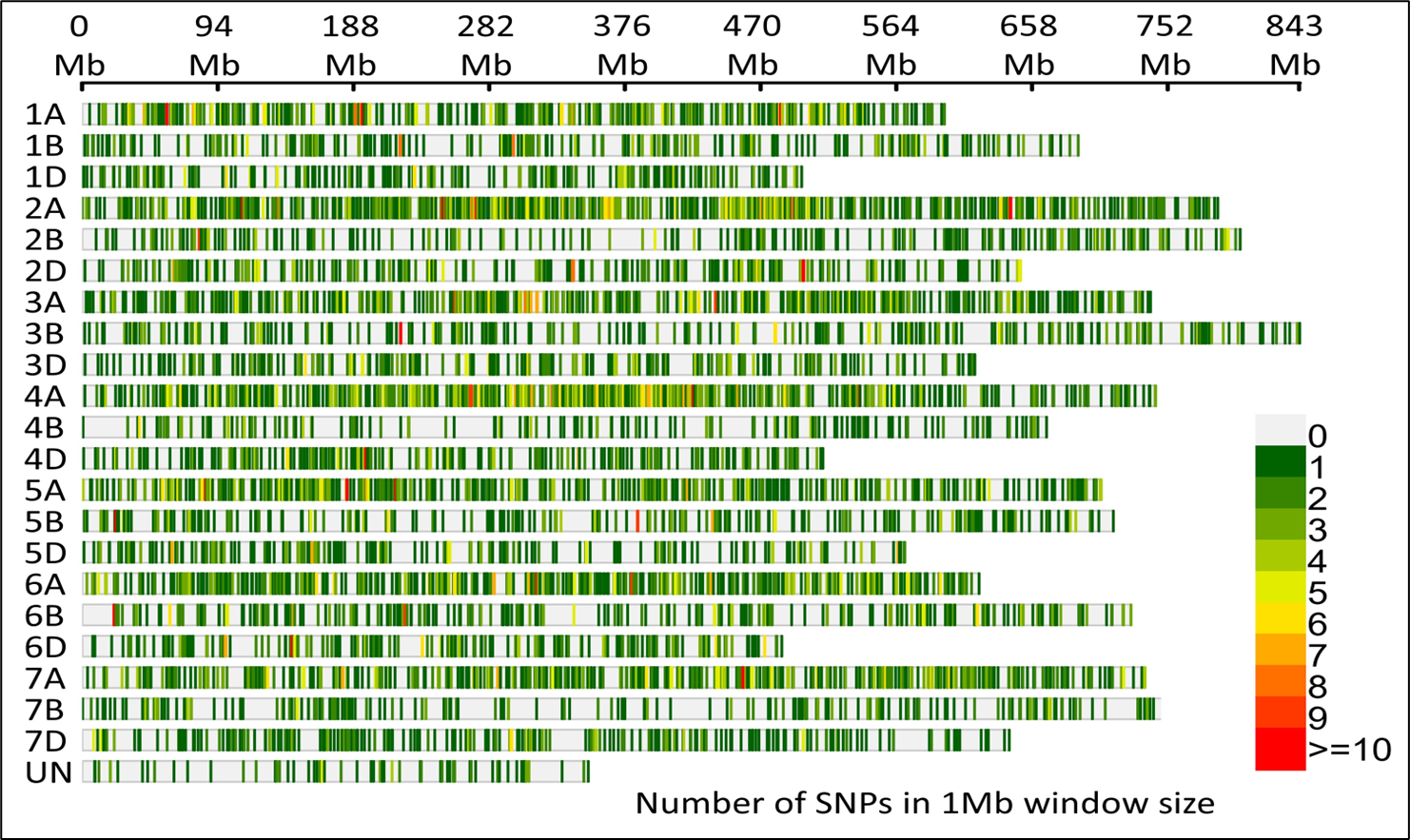


**Supplemental Figure S4.** Density of SNP markers (n = 9,858 SNPs) per 1Mb window on wheat chromosomes in a set of 459 hard winter wheat genotypes selected from the 2021 and 2022 NRPN, SRPN, and RGON.


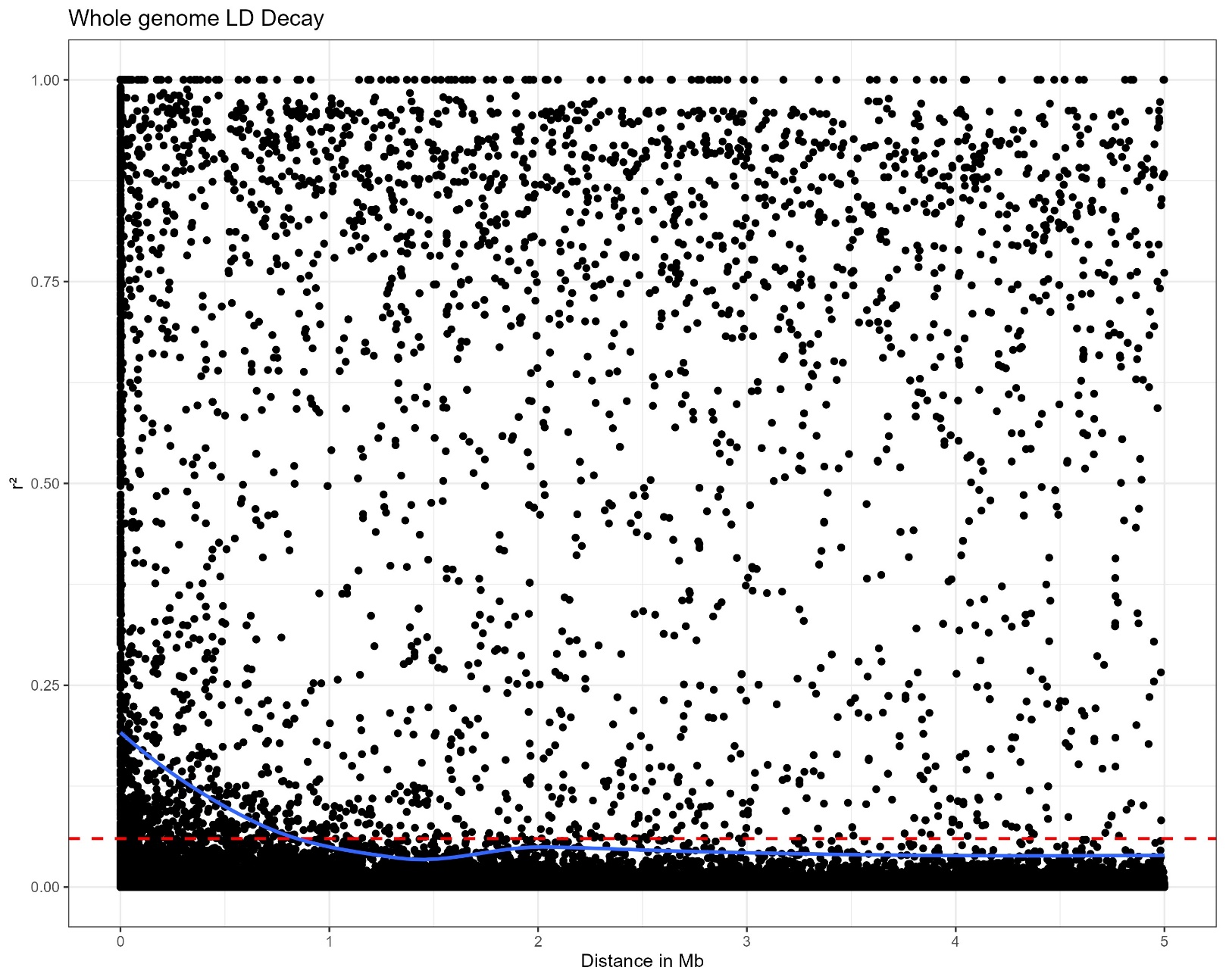


**Supplemental Figure S5.** Scatter plot showing linkage disequilibrium (LD) decay across the genome. The LD estimates (*r*^2^) for pairs of SNPs were plotted against the corresponding physical positions in million base pair (Mb) based on Chinese Spring wheat reference genome IWGSC_RefSeqv2.1 (Zhu et al., 2021).


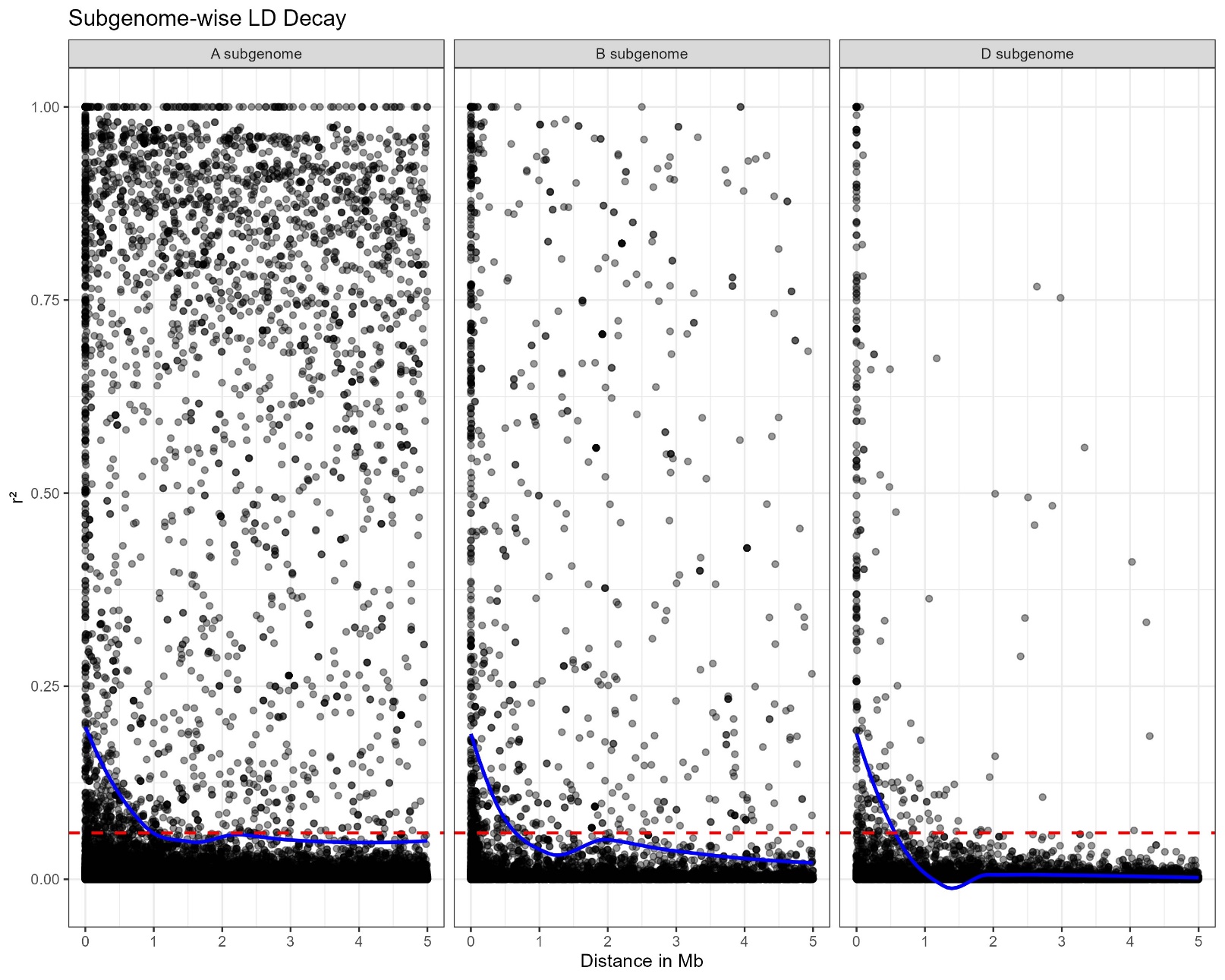


**Supplemental Figure S6.** Scatter plot showing linkage disequilibrium (LD) decay in sub genomes A, B, and D. The LD estimates (*r*^2^) for pairs of SNPs were plotted against the corresponding physical positions in million base pair (Mb) based on Chinese Spring wheat reference genome IWGSC_RefSeqv2.1 (Zhu et al., 2021).


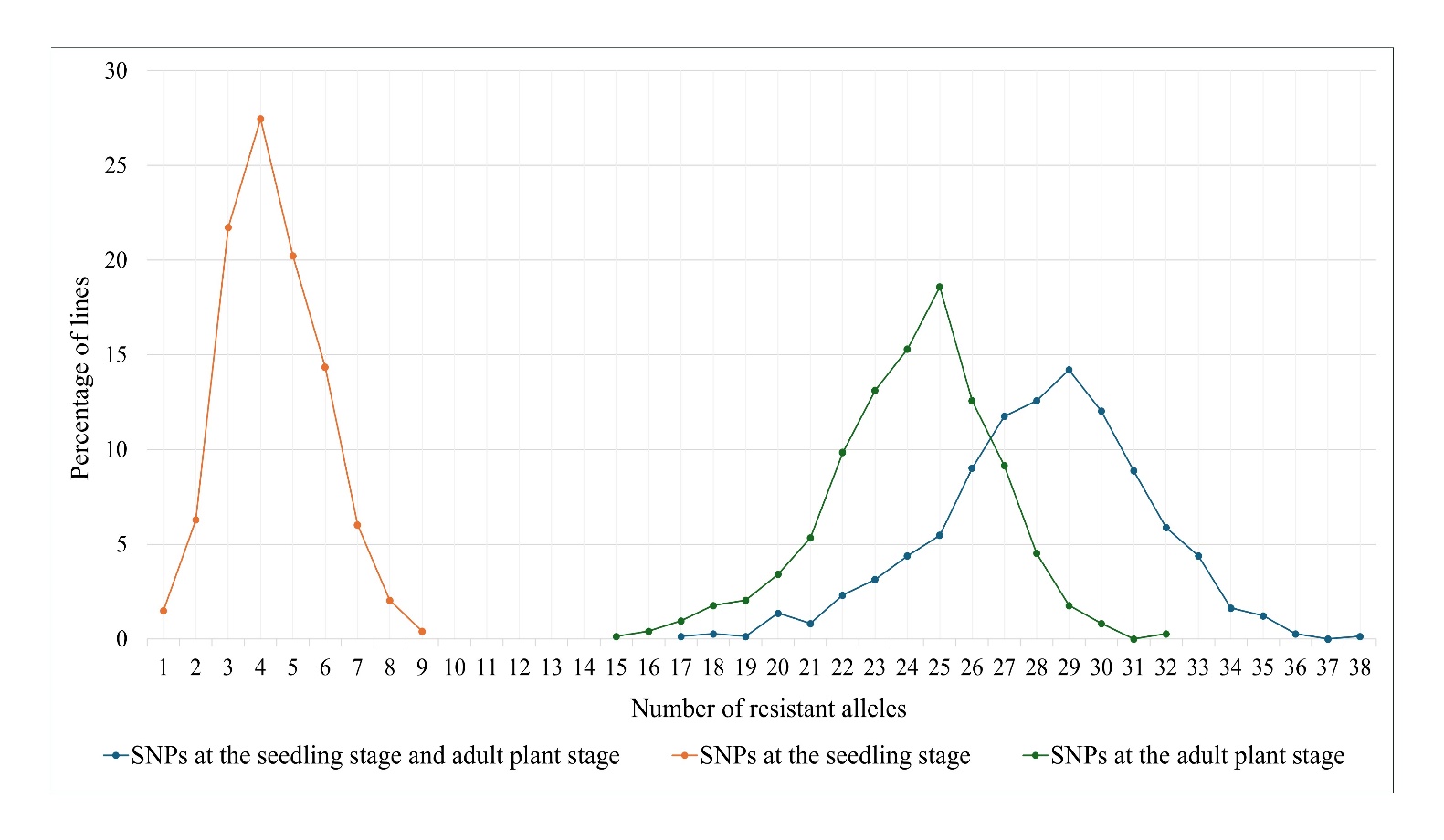
**Supplemental Figure S7.** Percentages of 732 wheat genotypes originated from the 2021 and 2022 NRPN, SRPN, and RGON carrying different number of resistant alleles of the 59 significant SNPs associated with leaf rust response. 20 SNPs were associated with leaf rust response at the seedling stage and 42 SNPs were associated with leaf rust response at the adult plant stage.


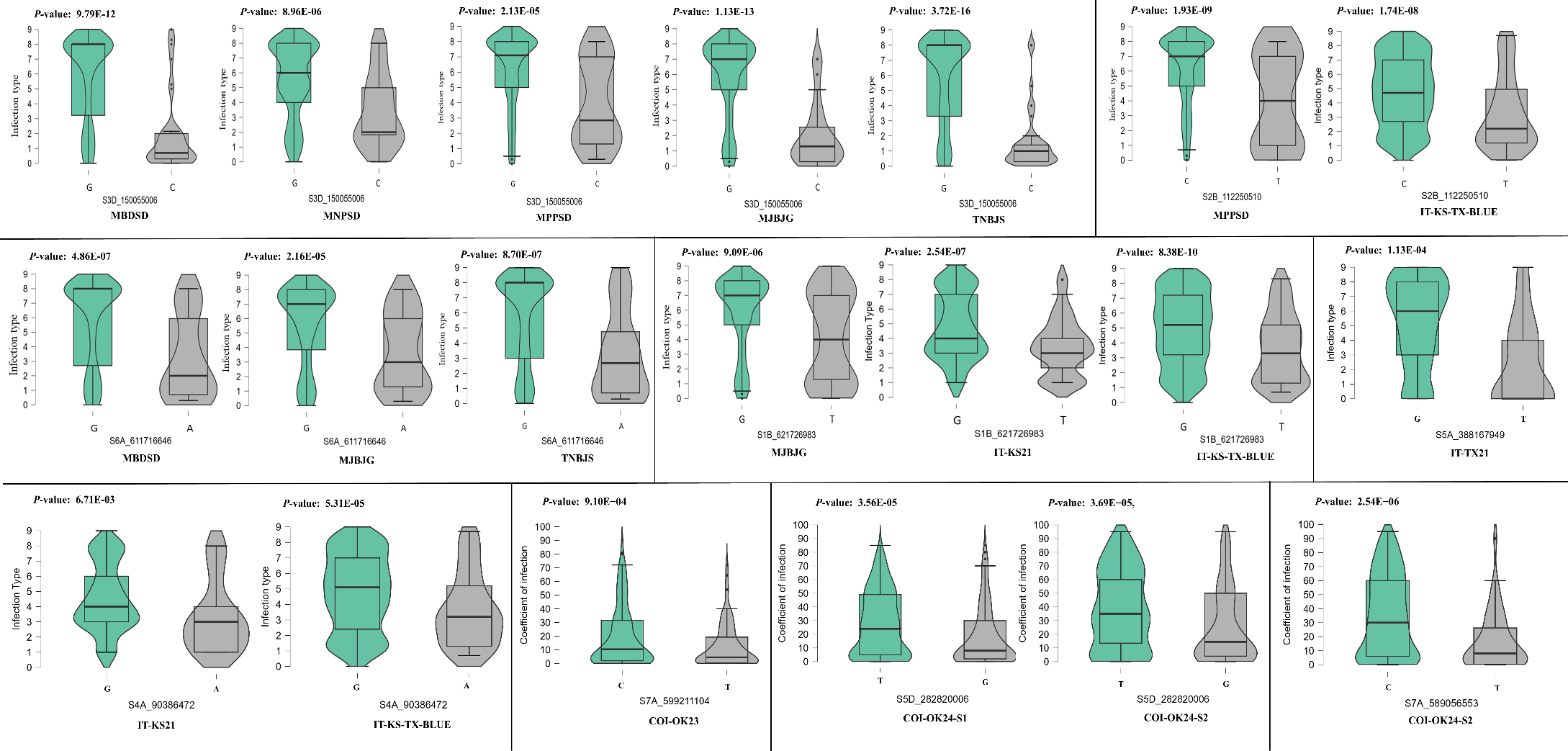


**Supplemental Figure S8.** Violin plots show leaf rust response distributions of genotypes carrying resistant and susceptible alleles of nine SNPs that can be useful for marker-assisted selection. *P* values in this figure indicate two-sided Welch t-test *P* values. The five *P. triticna* races MNPSD, MPPSD, TNBJS, MBDSD, and MJBJG were tested at the seedling stage. COI-OK23: coefficient of infection of 2021 and 2022 NRPN, SRPN and RGON genotypes (n = 459) tested at the adult plant stage in Oklahoma in 2023; COI-OK24-S1 & COI-OK24-S2: first and second scoring of coefficient of infection of 2021 and 2022 NRPN, SRPN, and RGON genotypes (n = 459) tested at the adult plant stage in Oklahoma in 2024, respectively; IT-TX21: ITs of 2021 RGON genotypes (n = 300) tested at the adult plant stage in Texas in 2021; IT-KS21: ITs of 2021 NRPN, SRPN, and RGON genotypes (n = 377) tested at the adult plant stage in Kansas in 2021; IT-KS-TX-BLUE: best linear estimates for ITs of 681 genotypes (IT-KS-TX-BLUE) across three field environments IT-TX21, IT-TX22, and IT-KS21.
